# Estimating the Information Extracted by a Single Spiking Neuron from a Continuous Input Time Series

**DOI:** 10.3389/fncom.2017.00049

**Published:** 2017-06-15

**Authors:** Fleur Zeldenrust, Sicco de Knecht, Wytse J. Wadman, Sophie Denève, Boris Gutkin

**Affiliations:** ^1^Department of Neurophysiology, Faculty of Science, Donders Institute for Brain, Cognition and Behaviour, Radboud UniversityNijmegen, Netherlands; ^2^Cellular and Systems Neurobiology, Swammerdam Institute for Life Sciences, University of AmsterdamAmsterdam, Netherlands; ^3^Group for Neural Theory, Institut National de la Santé et de la Recherche Médicale U960, Institute of Cognitive Studies, École Normale SupérieureParis, France; ^4^Department of Psychology, Center for Cognition and Decision Making, National Research University Higher School of EconomicsMoscow, Russia

**Keywords:** neural information processing, artificial neural network, *in vitro* electrophysiology, Bayesian neuron model, information theory

## Abstract

Understanding the relation between (sensory) stimuli and the activity of neurons (i.e., “the neural code”) lies at heart of understanding the computational properties of the brain. However, quantifying the information between a stimulus and a spike train has proven to be challenging. We propose a new (*in vitro*) method to measure how much information a single neuron transfers from the input it receives to its output spike train. The input is generated by an artificial neural network that responds to a randomly appearing and disappearing “sensory stimulus”: the hidden state. The sum of this network activity is injected as current input into the neuron under investigation. The mutual information between the hidden state on the one hand and spike trains of the artificial network or the recorded spike train on the other hand can easily be estimated due to the binary shape of the hidden state. The characteristics of the input current, such as the time constant as a result of the (dis)appearance rate of the hidden state or the amplitude of the input current (the firing frequency of the neurons in the artificial network), can independently be varied. As an example, we apply this method to pyramidal neurons in the CA1 of mouse hippocampi and compare the recorded spike trains to the optimal response of the “Bayesian neuron” (BN). We conclude that like in the BN, information transfer in hippocampal pyramidal cells is non-linear and amplifying: the information loss between the artificial input and the output spike train is high if the input to the neuron (the firing of the artificial network) is not very informative about the hidden state. If the input to the neuron does contain a lot of information about the hidden state, the information loss is low. Moreover, neurons increase their firing rates in case the (dis)appearance rate is high, so that the (relative) amount of transferred information stays constant.

## 1. Introduction

Neuroscientists aim to understand how the brain represents and transforms incoming information by quantifying the relation between (sensory) stimuli and the activity of neurons (i.e., “the neural code”). When researching such information transfer properties of neural systems, and in particular of single neurons, there are two main questions: (1) *what* information is encoded by a neuron (and what information is discarded), and (2) *how much* information is transferred (or lost). The first question is often investigated by fitting functional filter models such as a Linear–Non-linear Poisson model (Chichilnisky, 2001) or a Generalized Linear Model (Paninski, 2004) to the neural input and output (for an overview see Simoncelli et al., 2004; Schwartz et al., 2006). Here, we will focus on the second question: How much information is transferred by single neurons? This question was first posed by MacKay and McCulloch (1952) and de Ruyter van Steveninck and Bialek (1988) were first to develop a way to measure the information transfer in neurons. This quantitative approach to information transfer is important, because it shows how information transfer properties change. For instance, the amount of information a neuron transmits depends on the background activity of the network a neuron is embedded in Panzeri et al. (1999) and Shadlen and Newsome (1998), on neuromodulators such as dopamine (Cruz et al., 2011) and on the type of code that is used (i.e., a “temporal” or “rate” code, Panzeri et al., 2001).

Researchers have attempted to measure the information transfer from presynaptic activity to output spike trains in neurons in different experimental setups and sensory systems *in vivo* and *in vitro* (including the visual system of the fly (de Ruyter van Steveninck and Bialek, 1988) and the whisker system of rats (Panzeri et al., 2001), using different information theoretical measures (for an overview, see Borst and Theunissen, 1999; Dimitrov et al., 2011). However, quantifying the information between a stimulus and a spike train has proven to be challenging. For example, information can be measured by reconstructing the stimulus from a spike train, and estimating the signal-to-noise ratio (Bialek et al., 1991; Rieke et al., 1997). This method requires a large amount of data, since a model needs to be fitted to the neural response (e.g., a linear filter and transfer function) before transferred information can be measured. Alternatively, information can be measured using the so-called “direct method” (de Ruyter van Steveninck et al., 1997; Strong et al., 1998), in which the response variability is used to estimate the mutual information between stimulus and spike train output. Measuring the information between a neuron's input and output this way involves various difficulties and biases, including the need to repeat a stimulus many times (or for a vary long time) and a bias due to limited sample sizes (Treves and Panzeri, 1995; Strong et al., 1998). Moreover, it might be difficult to determine what kind of stimulus to use, and in these setups the stimulus and the measured neuron are often several synapses away, making it difficult to assess where a measured loss of information happens. Finally, the choice of what set of stimuli to use is non-trivial.

Here we present a method to estimate how much information is contained in the spike train of a single neuron in an *in vitro* setup. The neuron is presented with an current input, generated by a population of artificial presynaptic neurons that respond to a randomly appearing and disappearing preferred stimulus: the hidden state (Denève, 2008a; Lochmann and Denève, 2008). This hidden state mimicks for instance a randomly appearing bar with a preferred orientation (for cells in primary visual cortex) or sound with a preferred frequency (for cells in auditory cortex). The information estimate is calculated by comparing the absence/presence of the hidden state and an estimate of the presence of this stimulus, based on the output spike train. The method does not require vast amounts of data or many repetitions. The method can be applied in any *in vitro* setup (so it not limited to sensory systems). Moreover, various experimental parameters such as the autocorrelation time-constant due to the (dis)appearance rate of the hidden state or the specific amount of information in the input and the amplitude of the signal relative to the background noise can systematically be varied, while the input is still close to the natural stimuli neurons normally receive. Finally, since we have a model of the optimal response (the “Bayesian neuron,” Denève, 2008a), the quality of the performance of the neuron can be rigorously assessed.

The goal of the method presented here is to define an experimental paradigm with which the information (loss) of the spike-generating process can be quantified and compared (for instance between neuropharmacological states) in an *in vitro* paradigm. This information-calculation is based on previous work (Denève, 2008a; Lochmann and Denève, 2008), where a similar method was used to compare single-compartment models. Here, we add the following to the existing method: Firstly, we replace delta-spikes by exponential kernels to mimick Post-Synaptic Currents (PSCs). Secondly, we define the output of the artificial neural network as a current output, and scale it so that it can be injected in a current-clamp setup. Thirdly, we show that the mutual information in the input current can be kept constant while varying experimental parameters. There is a a trade-off between the autocorrelation time and the firing rates of the artificial presynaptic neurons: if the autocorrelation time is short (i.e., the hidden state appears and disappears with a high rate), a high firing rate of the presynaptic neurons is needed to keep the information in the input current constant^1^. Finally, we provide an example of an *in vitro* experiment where this paradigm is used. We apply the method presented here to pyramidal neurons in region CA1 of the rat hippocampus in an *in vitro* slice, to quantify the information loss from input to output spike train as a function of the stimulus (dis)appearance rate, the input current amplitude, and the information content of the input current (for an overview of other coding properties of these cells, see Hasselmo, 2011).

## 2. Methods

Here we present an experimental method to estimate how much information is contained in the spike train of a single neuron. In the first part of this methods section, we summarize and explain the theory behind the method. In order to easily estimate the information in a spike train, the neuron has to respond to a special type of input generated by an artificial neural network, which is explained first in Section 2.1.1. In the next Section 2.1.2, we explain how this special form of a noisy input can be used to quantify the information in the output spike train. The theoretical derivation follows Lochmann and Denève (2008), who compared model-neurons this way. Next, we define an optimal response model (Denève, 2008a; Section 2.1.3), which sets a benchmark for the performance of the recorded neuron.

In the second part of the methods section, we zoom in on the experimental part of the method: in Section 2.2.1 we explain how the activity of the artificial neural network, which is in arbitrary units, can be scaled so that it can be used as a fluctuating current input in an *in vitro* setup. Next, the input parameters used in the experiments are summarized (Section 2.2.2). Finally, the details of the experimental slice preparation and recording are given (Section 2.2.3).

### 2.1. Theory

#### 2.1.1. Input generation

**Figure 1:**
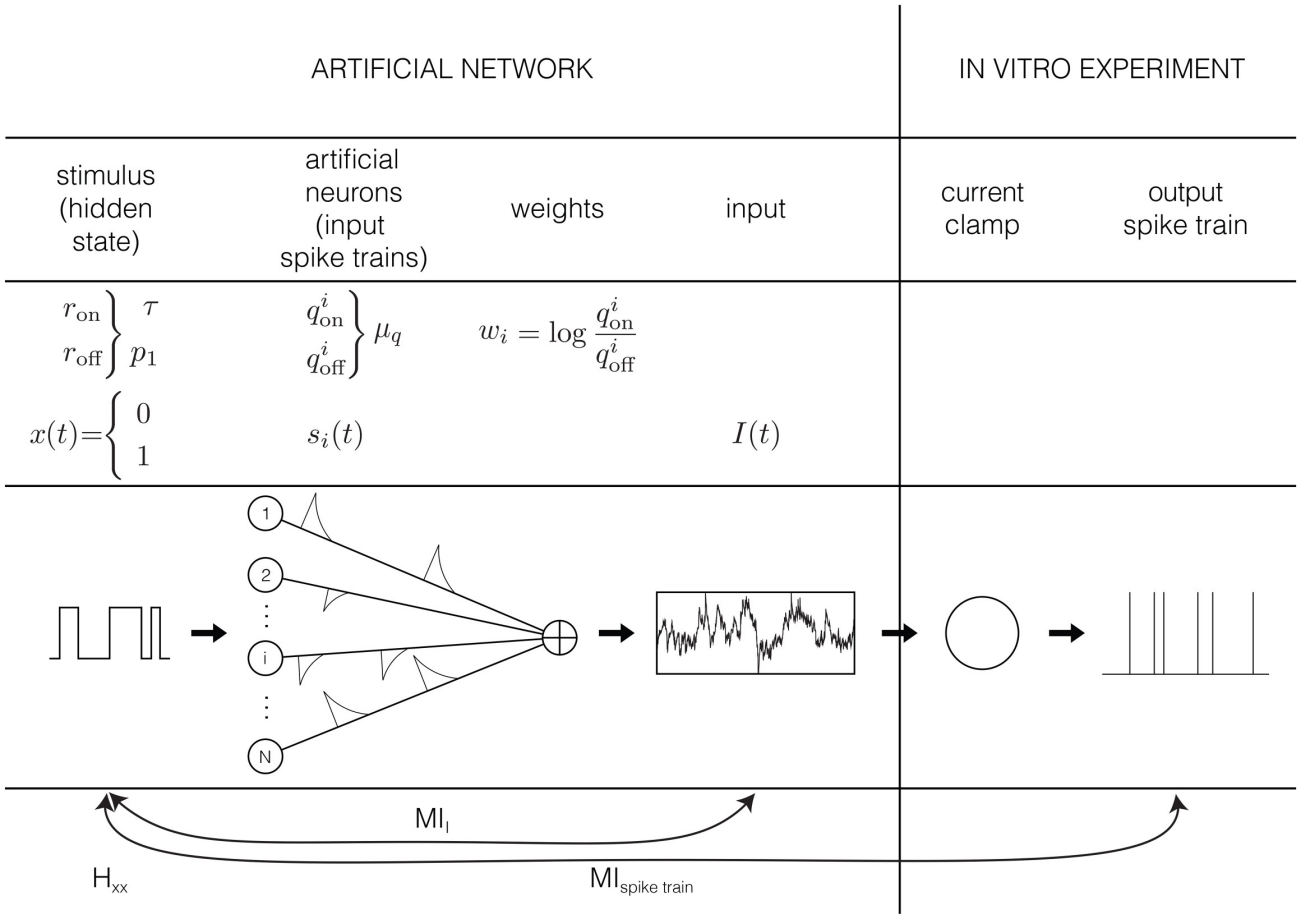
Graphical representation of the input model: the input is generated by *N* artificial neurons *i* that fire Poisson spike trains with rates $q_{\text{on}}^{i}$ and $q_{\text{off}}^{i}$ in response to a hidden Markov model, with rates *r*_on_ and *r*_off_. The spikes of each artificial neuron are convolved with an exponential kernel with a time constant of 5 ms and a weight of $w_{i}=\log\frac{q_{\text{on}}^{i}}{q_{\text{off}}^{i}}$. This current is injected into hippocampal pyramidal cells in an *in vitro* current clamp setup. The resulting spike trains are recorded and used to reconstruct the hidden state.

Except for sensory receptors, neurons in the brain respond to input generated by other neurons. We assume here that neurons respond to the absence or presence of a preferred stimulus feature, for instance an edge in a preferred orientation (visual system). This absence or presence of the preferred stimulus feature is represented by the hidden state *x* (Figure 1): a binary variable that equals 1 if the preferred stimulus is present, and 0 if it is absemt. We assume that this preferred stimulus appears and disappears randomly following a memoryless (Markov) process with rates *r*_on_ and *r*_off_. Or, stated differently, the quantities $\tau \;=\;\frac{1}{r_{\text{on}}+r_{\text{off}}}$ and $p_{1}=\frac{r_{\text{on}}}{r_{\text{on}}+r_{\text{off}}}$ quantify respectively how fast the hidden state switches and the probability of finding the hidden state in the “ON” (1) state.

The second assumption in the input generation, is that neurons do not directly observe the hidden state, but receive synaptic inputs from a population of *N* presynaptic neurons *i*, whose firing rate is modulated by the stimulus so that each fire Poisson spike trains with rate $q_{\text{on}}^{i}$ when *x* = 1, and $q_{\text{off}}^{i}$ when *x* = 0. These two assumptions are comparable to the assumptions that are implicitly made when estimating tuning curves, for instance by fitting filter models such as a Linear-Nonlinear Poisson model (Chichilnisky, 2001) to sensory stimuli: in both cases it is assumed that a neuron responds only to the present value (so no history or reverberation effects) of a preferred stimulus feature that it does not have direct access to.

Each of the spikes from the population of artificial presynaptic neurons is convolved with an exponential kernel with a time constant of 5 ms and a unitary surface. Moreover, the spike trains from different presynaptic neurons are weighted according to their reliability, i.e., $w_{i}=\log\frac{q_{\text{on}}^{i}}{q_{\text{off}}^{i}}$ (Figure 1). This is the third and strongest assumption of the input generation. These values for the weights result in an optimally informative total input current (Denève, 2008a), and can be learned with an unsupervised, local, spike-dependent learning rule (Denève, 2008b). We did not use a learning rule here, but just used the “optimal” weights. The relation between the weights and the firing frequencies makes sense intuitively: we assume that the neuron listens strongly to informative neurons ($q_{\text{on}}^{i}>>q_{\text{off}}^{i}$, results in *w*_*i*_ >> 0), not to neurons that are not informative ($q_{\text{on}}^{i}\approx q_{\text{off}}^{i}$, so *w*_*i*_ ≈ 0) and neurons that fire more when the preferred stimulus is absent have an inhibitory contribution ($q_{\text{on}}^{i}<<q_{\text{off}}^{i}$, results in *w*_*i*_ << 0). Given these weights, the sum-total synaptic input is given by

(1) $$\begin{array}{l} I=\overset{N}{\underset{i=1}{\sum }}w_{i}s_{i}*k, \end{array}$$

where * denotes a convolution with the exponential kernel *k*(*t*) and $s_{i}=\overset{M_{i}}{\underset{m_{i}=1}{\sum }}\delta \left(t-t_{m_{i}}\right)$ is the spike train of artificial neuron *i* that depends on the hidden state through $q_{\text{on}}^{i}$ and $q_{\text{off}}^{i}$. However, this input cannot be injected directly into a neuron in an *in vitro* setup or into a model neuron yet: it has to be scaled from dimensionless units to ampère A, which will be explained in Section 2.2.1. The autocorrelation time constant of the input depends on the switching rates of the hidden state (through $\tau =\frac{1}{r_{\text{on}}\;+\;r_{\text{off}}}$ and on the distribution of firing rates in the artificial neural network $q_{\text{on}}^{i}$ and $q_{\text{off}}^{i}$. Since we do not know anything a priori about the distributions of the firing rates $q_{\text{on}}^{i}$ and $q_{\text{off}}^{i}$, we make the most simple assumption and draw them from a Gaussian distribution. So $q_{\text{on}}^{i}$ and $q_{\text{off}}^{i}$ are all drawn from a Gaussian distribution with mean μ_*q*_ and standard deviation $\sigma_{q}=\sqrt{\frac{1}{8}}\mu_{q}$ (the value if σ_*q*_ is chosen so that virtually all firing rates are positive). Note that even though the firing rates $q_{\text{on}}^{i}$ and $q_{\text{off}}^{i}$ are drawn from the same distribution, this generally does not mean they have the same value.

#### 2.1.2. Estimating mutual information

The mutual information between the hidden state and the the input (*MI*_*I*_) or any output spike train (*MI*_spike train_) in response to this input can easily be estimated, because the input defined in the previous section uses a hidden state *x*. In this section, we will explain how to estimate this information in a spike train (the method can be applied to any spike train, be it recorded, simulated any other spike train). We start by estimating the entropy of the hidden state. Next, we consider the following two steps: (1) the transformation from hidden state to input (*MI*_*I*_), and (2) the transformation from input to spike train (i.e., the neural spike generating process, *MI*_spike train_). By definition, *MI*_*I*_ and *MI*_spike train_ cannot exceed the entropy of the hidden state *H*_*xx*_ (determined by *p*_1_, Equation 4). If there would be no information loss, the mutual information between the spike train and the hidden state equals the entropy of the hidden state: *MI*_spike train_ = *MI*_*I*_ = *H*_*xx*_. However, in practice every step will result in information loss: *MI*_spike train_ < *MI*_*I*_ < *H*_*xx*_. Since we have access to *MI*_spike train_, *MI*_*I*_, and *H*_*xx*_, we can estimate the information loss at every step.

The derivation follows Lochmann and Denève (2008) and Denève (2008a). The method requires two assumptions; firstly an ergodic argument: it is assumed that an average over samples can be replaced by an average over time. This means that if in an experiment the setup is not stationary during the time window for which the mutual information is calculated, the approximation fails. Secondly, it is assumed that output spike trains are by approximation Poissonian. The estimate of the mutual information is not strongly sensitive to this assumption, but strong deviations from Poissonian statistics will make the estimate fail. Time is measured in discrete steps, as most simulations and experiments use finite sampling rates. The mutual information is estimated for a single time-step, so it is an information rate (in bits/second). However, for simplicity and since we do not adjust the time step of our simulations of experiments here, we will only report the mutual information (in bits).

In Section 3, we will often use the fraction of transferred entropy

(2) $$\begin{array}{l} F={\langle \frac{\hat{M}I}{{Ĥ}_{xx}}\rangle }_{\text{samples or simulations}}, \end{array}$$

where the brackets denote an average over samples orsimulations. This fraction shows how much of the entropy of the hidden state is transferred to the output spike train, and should therefore always have a value between 0 (since information or entropy cannot become negative) and 1 (the mutual information should never exceed the entropy of the hidden state). Similarly, we will use the fraction of transferred information

(3) $$\begin{array}{l} FI=\frac{{\hat{M}I}_{\text{spike train}}}{{\hat{M}I}_{I}}, \end{array}$$

which should also have a value between 0 (no information about the hidden state in the input was transferred to the output spike train) and 1 (all information in the input was transferred to the output spike train).

##### 2.1.2.1. Entropy of the hidden state

The theoretical value of entropy of the hidden state on each moment in time depends only on the probability that the hidden state is 1 (because a Markov process is memoryless):

(4) $$\begin{array}{l} H_{xx}=-p_{1}\log_{2}\left(p_{1}\right)-\left(1-p_{1}\right)\log_{2}\left(1-p_{1}\right). \end{array}$$

However, for a given realization (the full sequence of hidden state values up to time *t*: *x*_0 → *t*_), the estimate of the entropy:

(5) $$\begin{array}{lll} {Ĥ}_{xx} & = & -{\langle x_{0\to t}\rangle }_{\text{time}}\log_{2}\left({\langle x_{0\to t}\rangle }_{\text{time}}\right)\\ -\;\left(1-{\langle x_{0\to t}\rangle }_{\text{time}}\right)\log_{2}\left(1-{\langle x_{0\to t}\rangle }_{\text{time}}\right). \end{array}$$

could show small deviations from its true value given in Equation (4).

##### 2.1.2.2. Conditional entropy

We start with a general estimate of the mutual information between the hidden state *x* and either an output spike train or the input. We use *y* to denote the history of the spike train or the input until now, and *Y* as the set of values *y* can take. The estimated mutual information *MI* is defined as the difference between the estimate of the entropy of the hidden state and the estimate of the conditional entropy of the hidden state given the history of *y* (spike train or input) until now:

(6) $$\begin{array}{l} \hat{M}I={Ĥ}_{xx}-{Ĥ}_{xy}. \end{array}$$

The conditional entropy of *x* given *y* is defined by

(7) $$\begin{array}{l} \begin{array}{cc} H_{xy} & =-\underset{x\in X,y\in Y}{\sum }p\left(x,y\right)\log_{2}\left(p\left(x|y\right)\right), \end{array} \end{array}$$

where *X* is the set of values *x* can take (i.e., *X* = {0, 1}). Since the hidden state can only take the values 0 and 1, we can estimate the conditional entropy by averaging over time:

(8) $$\begin{array}{l} {\hat{H}}_{xy}=-{\langle x\log_{2}(p(x=1|y))+(1-x)\log_{2}(p(x=0|y))\rangle }_{\text{time}}\\ \;=-\langle x\log_{2}(p(x=1|y))\\ \;+\;(1-x)\log_{2}((1-p(x=1|y)))\rangle_{\text{time}}, \end{array}$$

where we used the ergodic argument mentioned before to approximate an average over samples by an average over time. In the following two sections, we will explain how to estimate *p*(*x* = 1|*y*) and *p*(*x* = 0|*y*) based on either the input or an output spike train. Remember that *x* denotes the *current* value of the hidden state, whereas *y* signifies the spike train or input *history* up until now.

##### 2.1.2.3. Mutual information between the hidden state and the input

To estimate of the conditional entropy of the hidden state given the input history, we have to estimate the probability of the hidden state being equal to 1 given the history of the input. Following the derivation in Denève (2008a), *L*(*t*), the temporal evolution of the posterior log-likelihood of the hidden state being 1 based on the input history

(9) $$\begin{array}{l} L\left(t\right)=\log_{2}\frac{p\left(x=1|I_{0\to t}\right)}{p\left(x=0|I_{0\to t}\right)}=\log_{2}\frac{p\left(x=1|I_{0\to t}\right)}{1-p\left(x=1|I_{0\to t}\right)} \end{array}$$

can be estimated using the following differential equation:

(10) $$\begin{array}{l} \frac{d\hat{L}}{dt}=r_{\text{on}}\left(1+e^{-\hat{L}}\right)-r_{off}\left(1+e^{\hat{L}}\right)+I\left(t\right)-\theta , \end{array}$$

where $\theta =\overset{N}{\underset{i=1}{\sum }}q_{\text{on}}^{i}-q_{\text{off}}^{i}$ is the constant offset of the input, which is chosen to be equal to 0 in this paper^2^. So if we generate an input using the method from Section 2.1.1, we can integrate $\hat{L}$ using Equation (10) and estimate the mutual information using Equation (8) and the following estimate of the probability that the hidden state equals 1 given the input history:

(11) $$\begin{array}{l} \hat{p}\left(x=1|I_{0\to t}\right)=\frac{1}{1+e^{-\hat{L}}}. \end{array}$$

##### 2.1.2.4. Mutual information between the hidden state and a spike train

The conditional entropy and the mutual information between the hidden state and an output spike train $\rho \left(t\right)=\overset{M}{\underset{m=1}{\sum }}\delta \left(t-t_{m}\right)$ can be estimated using the same method as for estimating the mutual information between the hidden state and the input: by integrating the log-likelihood *L* over time. However, parameter *I* in Equation (10) should now be replaced by *I*_spike train_, generated with the help of Equation (1). In this equation, the exponential kernel *k* was used, because δ-spikes cannot be used in an experimental setup. However, for the information calculation, δ-spikes are not a problem, so the exponential kernel will be discarded^3^. For a given spike train, we need to estimate both θ and *w*, so we need to estimate *q*_on_ and *q*_off_:

(12) $$\begin{array}{l} \begin{array}{cc} {\hat{q}}_{\text{on}} & =\frac{\int_{t{|}_{x=1}}\rho \left(t\right)dt}{\int_{t{|}_{x=1}}dt}=\frac{\text{total \# spikes while }x=1}{\text{total time }x=1}\\ {\hat{q}}_{\text{off}} & =\frac{\int_{t{|}_{x=0}}\rho \left(t\right)dt}{\int_{t{|}_{x=0}}dt}=\frac{\text{total \# spikes while }x=0}{\text{total time }x=0}. \end{array} \end{array}$$

Now, we can generate *I*_spike train_ for calculating *L* using Equation (10) and estimating the mutual information using Equations (8) and (11).

##### 2.1.2.5. Hidden state estimate and mean-squared error

With the help of Equation (9), an estimate of the hidden state can be defined: because the hidden state can only take the values 0 and 1, and the estimate of the probability that the hidden state is equal to one $\hat{p}\left(x=1|I_{0\to t}\right)$ can only take values between 0 and 1, $\hat{p}\left(x=1|I_{0\to t}\right)$ can be viewed as an estimate of the hidden state:

(13) $$\begin{array}{l} \hat{x}\left(t\right)=\hat{p}\left(x=1|I_{0\to t}\right)=\frac{1}{1+e^{-\hat{L}\left(t\right)}}. \end{array}$$

This can be used to calculate another measure of how well a spike train represents the hidden state, the mean-squared error (MSE)^4^:

(14) $$\begin{array}{l} MSE=\frac{1}{N_{t}}\overset{N_{t}}{\underset{t=1}{\sum }}{\left({\hat{x}}_{t}-x_{t}\right)}^{2}, \end{array}$$

where we used discretized time. We can normalize this *MSE* by dividing it by the *MSE* of Poisson spike-trains with the same number of spikes

(15) $$\begin{array}{l} MSE_{P}=\frac{MSE_{\text{spike train}}}{{\langle MSE_{\text{Poisson spike train}}\rangle }_{\text{simulations}}}. \end{array}$$

This gives us a quantity that is around 1 when a spike train performs as well as a Poisson spike train (so when there is no information about the hidden state in the spike train) and vanishes when the hidden state can be perfectly inferred from the spike train (so the *MSE* vanishes). We can also normalize *MSE*_spike train_ by dividing it by the mean-squared error obtained with the input *MSE*_*I*_:

(16) $$\begin{array}{l} FMSE=\frac{MSE_{\text{spike train}}}{MSE_{I}}. \end{array}$$

This represents how much noise the spike process of the neuron adds to the estimate of the hidden state: if it equals 1 the error of the estimate based on the input has the same size as the error based on the spike train, and the neuron transmits all the information in the input perfectly.

##### 2.1.2.6. Delays

The theoretical form of the input was derived using Dirac-delta spikes (Denève, 2008a). Since an input consisting of delta spikes cannot be used in an experimental setup, we chose to convolve the input with exponential kernels, which mimics cortical PSC shapes. However, since an exponential kernel rises instantaneously, but decays slowly, this introduces a delay in the input relative to the hidden state. On the next level, any neuron that responds to this input will have a non-vanishing membrane time constant, resulting in a further delay. With this reasoning, each processing level adds a few ms delay to the representation of the hidden state. To separate the effects due to delays and other effects influencing the quality of the representation, we also calculate the mutual information between the hidden state and a shifted version of the input or spike train: we calculate the time-value peak of the cross-correlogram between the hidden state and the input/spike train, and shift the input/spike train by this amount. The mutual information resulting from this calculation will be denoted by MI^*^.

#### 2.1.3. Optimal response model

One of the advantages of creating an input using a hidden Markov model is that we have a model for an optimal response: the “Bayesian neuron” (Denève, 2008a). This model compares the log odds ratio of the stimulus (i.e., the log-likelihood of the hidden state being 1, see Equation 9) based on the input *L* with the log odds ratio based on the output spike train *G*, and keeps this difference small by spiking at appropriate times. This neuron only spikes if the likelihood of the hidden state being 1 based on the output spike train is lower than the likelihood of the hidden state being 1 based on the input, thereby only transferring “new” information and making efficient use of its output spikes:

(17) $$\begin{array}{l} \begin{array}{cc} \frac{dL}{dt} & =r_{\text{on}}\left(1+e^{-L}\right)-r_{off}\left(1+e^{L}\right)+I\left(t\right)-\theta \\ \frac{dG}{dt} & =r_{\text{on}}\left(1+e^{-G}\right)-r_{off}\left(1+e^{G}\right)\\ \text{if }L>G+\frac{\eta }{2}:\left\{ \begin{array}{cc} \; & \text{ a spike is fired}\\ \; & G\to G+\eta \end{array}\right. \end{array} \end{array}$$

For a given input, the only free parameter in this model is η, the reset and threshold condition which sets the output firing rate of the neuron. The mutual information between a spike train and the hidden state necessarily depends on the firing rate: if a neuron does not spike, the mutual information vanishes. To signal whether the hidden state switches on (or off), the neuron needs to fire at least one spike every on (or off) state. Ideally, the firing rate of a spike train is comparable to $\frac{1}{\tau }$. We use parameter η to match the firing rate of the neurons we measured and the firing rate of the Bayesian neuron, to be able to compare the two.

### 2.2. Experimental design

#### 2.2.1. Scaling

The input defined in Section 2.1.1 is dimensionless (the weights only give a relative contribution, scaled to how informative the artificial neuron is about the hidden state). Input currents used in *in vitro* experiments has either unit ampère A (current clamp), volt V (voltage clamp), or siemens S (dynamic clamp). Therefore, the dimensionless theoretical “input current” from the artificial network has to be scaled so that it can be injected into the neuron in a current clamp setup (so we will have to scale the input generated by the artificial network to ampère A).

(18) $$\begin{array}{l} I_{\text{injected}}=I_{\text{hold}}+I_{\text{scale}}I_{\text{Markov}}\left(t\right), \end{array}$$

where *I*_Markov_(*t*) is the dimensionless “current” defined by Equation (1). Finding *I*_hold_ and *I*_scale_ is not a trivial procedure: how “strong” an input current is for a neuron depends on its sensitivity to input current. This sensitivity can depend on several neuronal properties such as its excitability (rheobase, the steepness of the input-frequency curve), but also on the size of the neuron and the strength of the seal of the patch clamp. Here, we chose the following solution:

- **Offset:** In the current clamp measurements the membrane potential was adjusted by a feedback system that injects current (*I*_hold_), so that the membrane potential stabilized to a desired value (−65 mV) before the actual measurement was started. From then on the value *I*_hold_ was fixed.
- **Amplitude:** We used a probe input (see Section 3.1.2) to define the amplitude with which to scale all inputs for a given neuron: we tried factors *I*_scale_ (with a resolution of 250 pA, so 250, 500, 750, 1000, 1250 pA, etc.) to set the firing rate response of the neuron to about 12 Hz overall (about 20 Hz when *x* = 1).

#### 2.2.2. Parameters

Every parameter set {τ, *p*_1_, μ_*q*_} defines an input “regime.” We chose three “difficult” (i.e., low *MI*_*I*_) regimes: a “slow” (S) regime, with a small μ_*q*_ and a large τ; a “fast” (F) regime, with a large μ_*q*_ and a small τ, and a “probe” (P) regime in between with intermediate μ_*q*_ and τ. The probe served to determine the scaling of the input (previous Section 2.2.1). For comparison, we also used a “fast switching—low amplitude” (FL) regime with a very low information content and a “slow switching—high amplitude” (SH) regime with a high information content. The exact values and reasoning behind the regimes will be explained in Section 3.1.2.

As explained in the previous Section 2.2.1, the theoretical input generated by the artificial network needs to be scaled in order to use it in an experimental set-up. We scaled the inputs generated in the different regimes all with the same factor (Equation 18). From then on the value was fixed. To determine *I*_scale_ we used the probe (P) input, i.e., an input with the same information content as the S and F inputs, but with an intermediate τ:τ_probe_ and μ:μ_*q*,probe_. The mutual information between the hidden state and a spike train naturally depends on the firing rate. Therefore, we scale the input current so that each neuron responds with about the same firing rate to the probe input: about 12 Hz overall (about 20 Hz when *x* = 1).

The input defined in Section 2.1.1 was generated once for each regime, and consequently used as a “frozen noise” input for the experiments and simulations. The parameters for the generated input are shown in Table 1. In Section 3.1.2 we will motivate these choices. The input used in the experiments was 20 s for the probes, and 300 s for each of the other regimes. The mutual information was calculated on 15 consecutive windows of 20 s. Unless mentioned otherwise, we used a sampling rate of 5,000 Hz (so a time step of *dt* = 0.2 ms) for both the input in the experiments and the simulations. Due to the limited time we had for each neuron, we measured in each neuron both the “slow” (S) regime and the “fast” (F) regime, but only the “fast, low amplitude” (FL) OR “slow, high amplitude” (SH) regime in the following order: (1) F, (2) SH, (3) S or (1) S, (2) FL, (3) F. So the switching speed was always changed first, and the amplitude second.

**Table 1:** Parameter values for the different input regimes.

**Regime**	**Symbols**	**Abbreviations**	***r*_on_ (Hz)**	***r*_off_ (= 2 *r*_on_) (Hz)**	**τ (ms)**	**μ_*q*_ (Hz)**
Slow	•	S	6.7	13.3	50	0.5
Fast	▴	F	5*r*_on, slow_ = 33.3	66.7	10	5μ_*q*,slow_ = 2.5
Probe	■	P	2.5*r*_on, slow_ = 16.7	33.3	20	2.5μ_*q*, slow_ = 1.3
Slow, high amplitude	♦	SH	6.7	13.3	50	2.5
Fast, low amplitude	^*^	FL	33.3	66.7	10	0.5

We obtained valid recordings from 6 cells. We measured the mutual information of on traces of 20 s. Since we used 300 s recordings, this means we obtained 15 measurements of the mutual information per neuron and per regime.

#### 2.2.3. Experiments

##### 2.2.3.1. Animals and slice preparation

Electrophysiological experiments were performed using brain slices from 4 to 5 week old C57/Bl6 mice (Harlan, The Netherlands) of either sex (3 animals, 5 different slices in total). All experiments were performed with the approval of the committee on animal bioethics of the University of Amsterdam. Hippocampal acute slices were prepared in ice cold (4°C) modified artificial cerebro spinal fluid (ACSF, in mM)—120 choline Cl, 3.5 KCl, 0.5 CaCl_2_, 6 MgSO_4_, 1.25 NaH_2_PO_4_, 10 D-glucose, 25 NaHCO_3_. Animals were killed by decapitation, and 350 μm thick slices were cut in the horizontal plane on a vibrating slicer (Leica, VT1200S; Wetzlar, Germany). Slices were kept in a perfusion chamber with ACSF (in mM)—120 NaCl, 3.5 KCl, 2.5 CaCl_2_, 1.3 MgSO_4_, 1.25 NaH_2_PO_4_, 10 Glucose, 25 NaHCO_3_ at 32°C for 30 min, and then left at room temperature for at least 30 min until recordings started. For further details on the animals and slice preparation, see Wierenga and Wadman (2003).

##### 2.2.3.2. Electrophysiological recordings

Current-clamp recordings were made under constant superfusion of ACSF bubbled with carbogen (95% O_2_/5% CO_2_) at a temperature of 32°C. We recorded neurons solely from the pyramidal cell layer of region CA1 and identified the pyramidal cells using differential interference contrast (DIC) with a light source of 780 nm (Scientifica; Uckfield, UK), as well as on the basis of their firing properties. Neurons were recorded in whole cell current clamp configuration with the Axopatch 200B amplifier (Axon Instruments Inc.; Forster City, CA, USA). For these recordings we used a pipette solution with (in mM) 131.5 K-gluconate, 8.75 KCl, 10 HEPES, 0.5 EGTA, 4 MgATP, and 0.4 NaGTP, this solution was brought to a pH of 7.3. Glass pipettes with a resistance in the range of 2.5–4 MΩ were used. Signals were low-pass filtered at 5 kHz and sampled at 25 kHz. Series resistances was compensated up to 70%. Data was acquired with in-house MATLAB based routines (MathWorks, 2007b; Natick, MA, United States).

We compensated online for the liquid junction potential (14.5 mV), as calculated from the solutions. To determine *I*_hold_, we used a feedback system that stabilized the membrane potential to −65 mV until the actual measurement was started.

## 3. Results

In order to calculate the information transfer in single neurons in an *in vitro* setup, we designed an input current defined in Sections 2.1.1 and 2.2.1. Before we describe the results of the current clamp experiments, we will first discuss the properties of this input current.

### 3.1. Input properties

#### 3.1.1. Information in input depends on switching speed and firing rate

**Figure 2:**
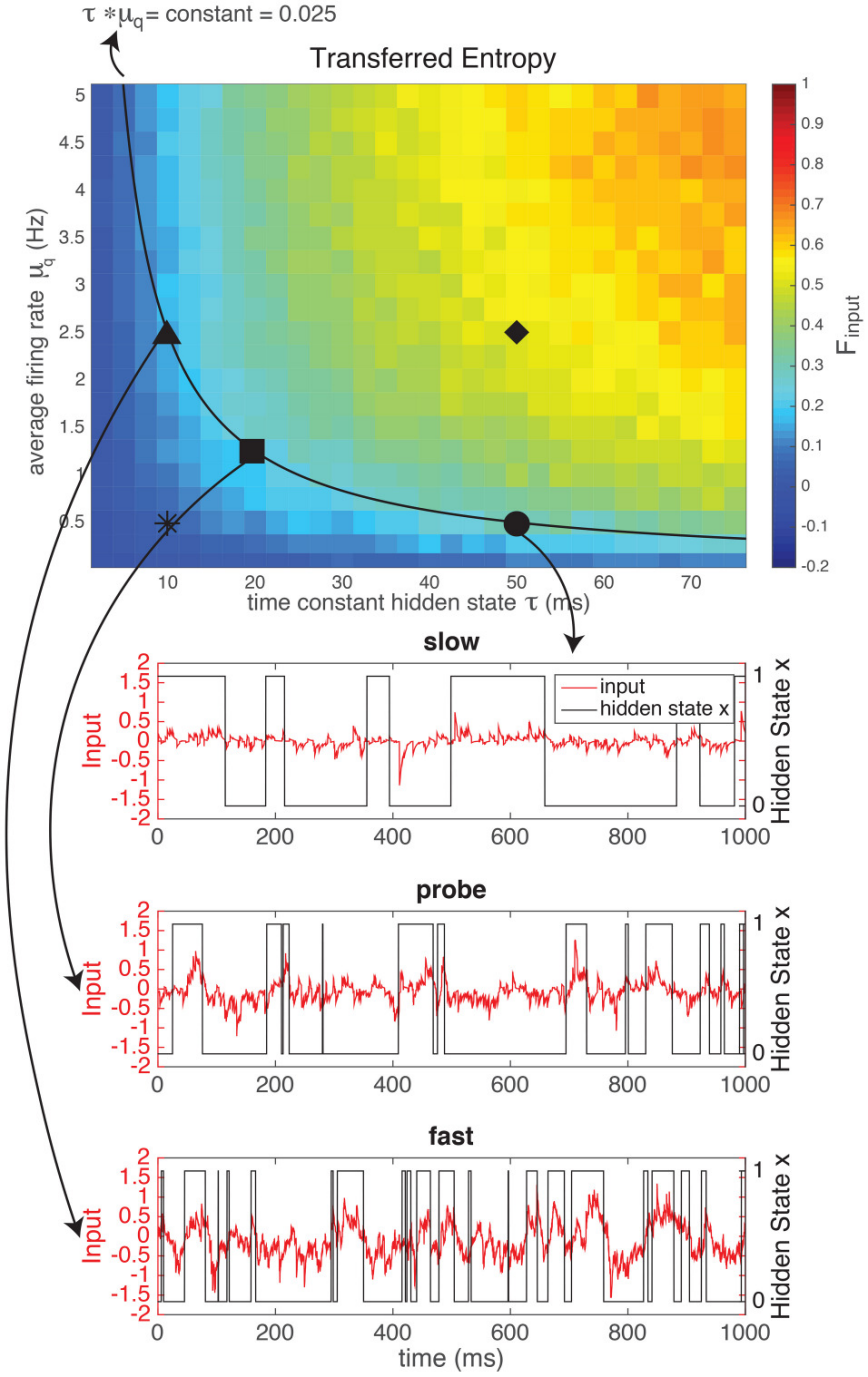
The mutual information between the hidden state and the input generated by a network of *N* = 1, 000 artificial neurons as a fraction of the entropy in the hidden state (top). This fraction of transferred information depends both on the firing rate of the neurons in the network (μ_*q*_) and on the switching speed (time constant τ) of the hidden state: if the hidden state switches faster, more spikes (i.e., higher firing rate or more neurons) are needed to reliably represent the hidden state in the input. For the experiments we chose three “difficult” (i.e., low information content) regimes: a “slow” regime (circle), with a low firing rate and a large τ, a “fast” regime (triangle), with a high firing rate and a small τ and a “probe” regime in between (square) with intermediate firing rates and τ, to determine the scaling of the input. For comparison, we also used a “fast switching—low amplitude” regime with a very low information content (star, example shown in Supplementary Material) and a “slow switching—high amplitude” regime with a high information content (diamond, example shown in Supplementary Material). NB Note that even though theoretically *MI* ≥ 0, due to our approximation $\hat{M}I$ can take small negative values. However, these effects are negligable (smallest value in this figure is $\hat{M}I=$ −0.0011).

The input defined by Equation (1), depends on the switching speed of the hidden state (*r*_on_ and *r*_off_) and on the firing rates of the artificial presynaptic neurons ($q_{\text{on}}^{i}$ and $q_{\text{off}}^{i}$, see Figure 1). The characteristics of the hidden state are external, i.e., they model how “the world outside of the animal” behaves. The characteristics of the artificial neurons model how neurons presynaptic to the real neuron (inside the animal) respond to the external stimulus. Both the external parameters of the “outside world” and the modeled internal parameters of the artificial neurons influence how much of the entropy of the hidden state (*H*_*xx*_ is transferred to the spike trains received by the neuron (mutual information in the input, *MI*_*I*_). In Figure 2 we kept the entropy of the hidden state constant (*r*_off_ = 2*r*_on_, so the probability of the hidden state being 1 equals $p_{1}=\frac{1}{3}$ and the entropy of the hidden state is *H*_*xx*_ ≈ 0.92 bits at each moment in time). The switching speed τ of the hidden state and the firing rates μ_*q*_ of the artificial presynaptic neurons were independently varied. We calculated the fraction of the entropy in the hidden state that gets transferred to the input (Equation 2). Figure 2 shows that there is a trade-off between the switching speed of the hidden state and the firing rates of the presynaptic neurons: if the switching speed is high (small τ), a high firing rate of the presynaptic neurons is needed to represent the hidden state, whereas for lower speeds the firing rates can be lower. This was expected: in order to represent *x*, one or more of spikes are needed to signal each period when *x* is in the “ON” state (i.e., a period when *x* = 1). A higher switching rate implies that these “ON” periods are shorter and more frequent. Even though the total “ON”-time might be unchanged, there are more separate “ON” states. Therefore, if every “ON”-state needs (at least) one output spike to be visible in the output spike train, more spikes are needed for a fast-switching hidden state (small τ). Note that since the artificial presynaptic neurons fire Poissonian spike trains, a higher overall firing rate can be obtained by either increasing the individual firing rates of the neurons (μ_*q*_), as in Figure 2, or by increasing the number of presynaptic neurons *N*^5^. The relationship between μ_*q*_ and τ is almost inversely proportional (black line shows inversely proportional relationship).

Even though the time constant of the hidden state (τ) and the firing rates of the presynaptic neurons (μ_*q*_) have a similar effect on the mutual information between the hidden state and the input, their effects on the shape of the input are quite different: the effect of increasing μ_*q*_ is to increase the amplitude of the input (Figure 2). Alternatively, increasing τ does not increase the amplitude, but changes the autocorrelation-time τ_auto_ (see Supplementary Material) of the input current signal. So, with τ and μ_*q*_ we can vary the input amplitude and autocorrelation-time independently, while keeping the mutual information between the input and the hidden state constant.

#### 3.1.2. Input regimes

In order to show the power of the method presented here, we designed two inputs with the same mutual information between the input current and the hidden state, but with a different amplitude (μ_*q*_) and time-constant (τ) on the basis of our results from the previous section. The results of the current clamp experiment will be shown in Section 3.2. Here, we explain the design of the experiment (Figure 2 and Table 1). We chose three “difficult” (i.e., low information content) regimes: a “slow” (S) regime (circle •), with a low amplitude and a large τ, a “fast” (F) regime (triangle ▴), with a high amplitude and a small τ, and a “probe” (P) regime in between (square ■) with intermediate firing rates and τ. The probe served to determine the scaling of the input current (Section 2.2.1). For comparison, we also used a “fast switching—low amplitude” (FL) regime with a very low information content (star *) and a “slow switching—high amplitude” (SH) regime with a high information content (diamond ♦).

As explained in Section 2.2.1, the theoretical input generated by the artificial network needs to be scaled in order to use it in an experimental set-up. We scaled the inputs from the different regimes all with the same factor (Equation 18). This factor was determined once for each neuron, from then on the value was fixed. To determine *I*_scale_ we used the probe (P) input defined before, i.e., an input with the same information content as the S and F inputs, but with an intermediate τ_probe_ and μ_*q*,probe_. As argued before, the mutual information between the hidden state and a spike train naturally depends on the firing rate. Therefore, we scale the input current so that each neuron responds with about the same firing rate to the probe input: about 12 Hz overall (about 20 Hz when *x* = 1).

### 3.2. Experimental results

#### 3.2.1. Representation of the hidden state by a single neuron

##### 3.2.1.1. Neurons perform a non-linear operation on their input

**Figure 3:**
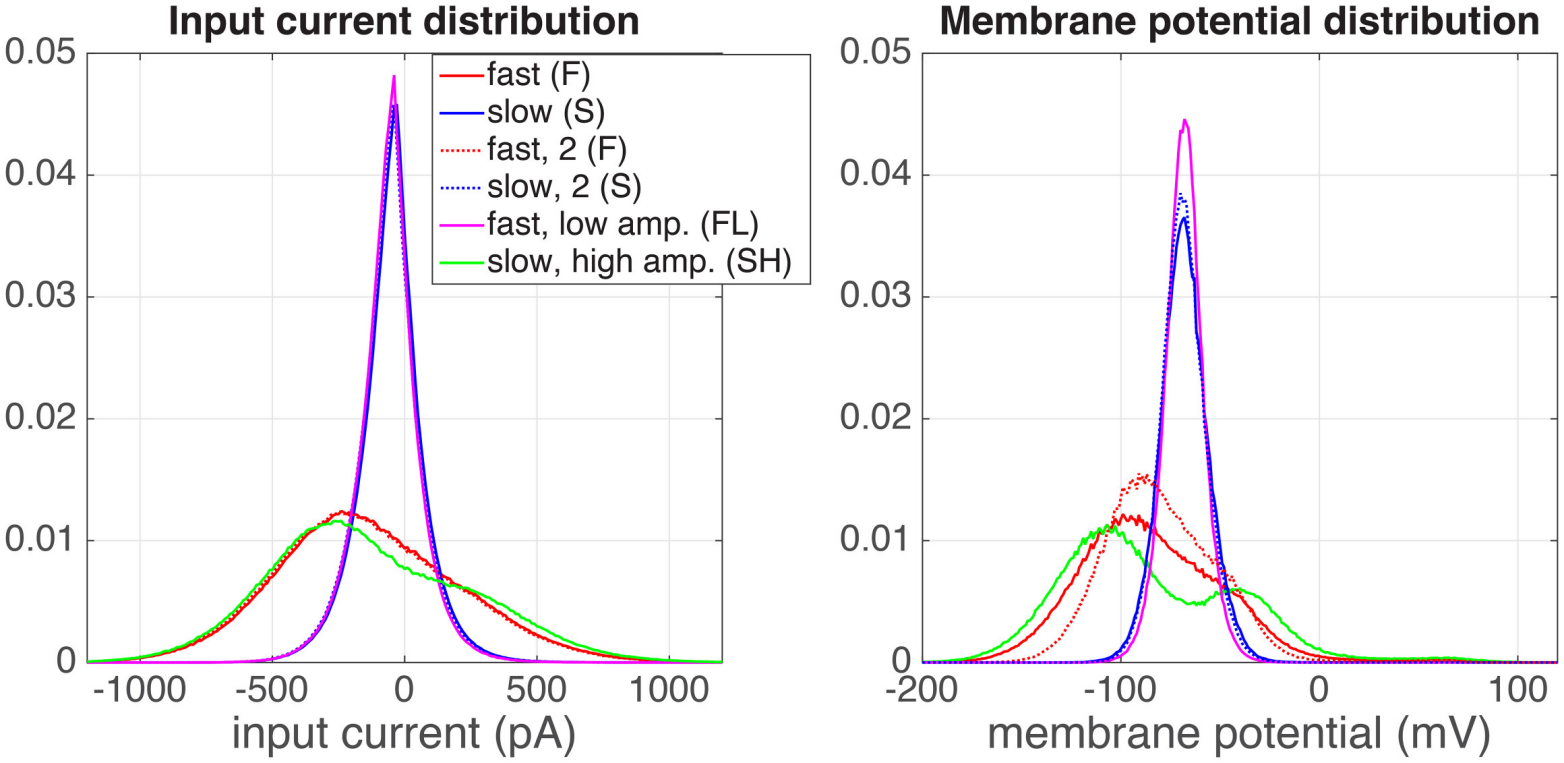
Distributions of the input current **(left)** and output membrane potential **(right)**, for a one of the recorded cells (depicted with symbol + in Figure 5). The experiment was performed in the following order of regimes: fast (red), slow, high amplitude (green), slow (blue), slow (blue, dotted), fast, low amplitude (pink), fast (red, dotted).

In the previous section, we explained the rationale behind the experiments. In Figure 3 we show the distributions of the injected input current (left) and the resulting membrane potential (right) of one example neuron (denoted with + in **Figure 5**). The input current distributions of both the S (blue), and the FL (pink) regimes were identical, as expected. There was a small difference between both F regimes (red) and the SH (green) regime, because in the F regime the “ON” state (*x* = 1) and “OFF” state (*x* = 0) are blurred by the exponential shape of the artificial EPSCs (Section 2.1.1). The resulting membrane potential distributions (Figure 3, right) are unimodal for both the S and the FL regimes, as expected. However, in the SH regime the (output) membrane potential distribution (green) is bimodal, whereas the (input) current distribution is unimodal for this regime (this effect was found for all cells for which we measured the SH regime). This means that the neuron performs a non-linear operation on the input current; with a linear transformation, the shape of the distribution would stay identical. Moreover, the distributions of the membrane potentials in both F regimes (red, full and dotted line) are not identical. This could be due to neural adaptation to the input or to non-stationary experimental conditions (for instance resistance of the seal with the pipette).

##### 3.2.1.2. Neurons transmit information about the hidden state

**Figure 4:**
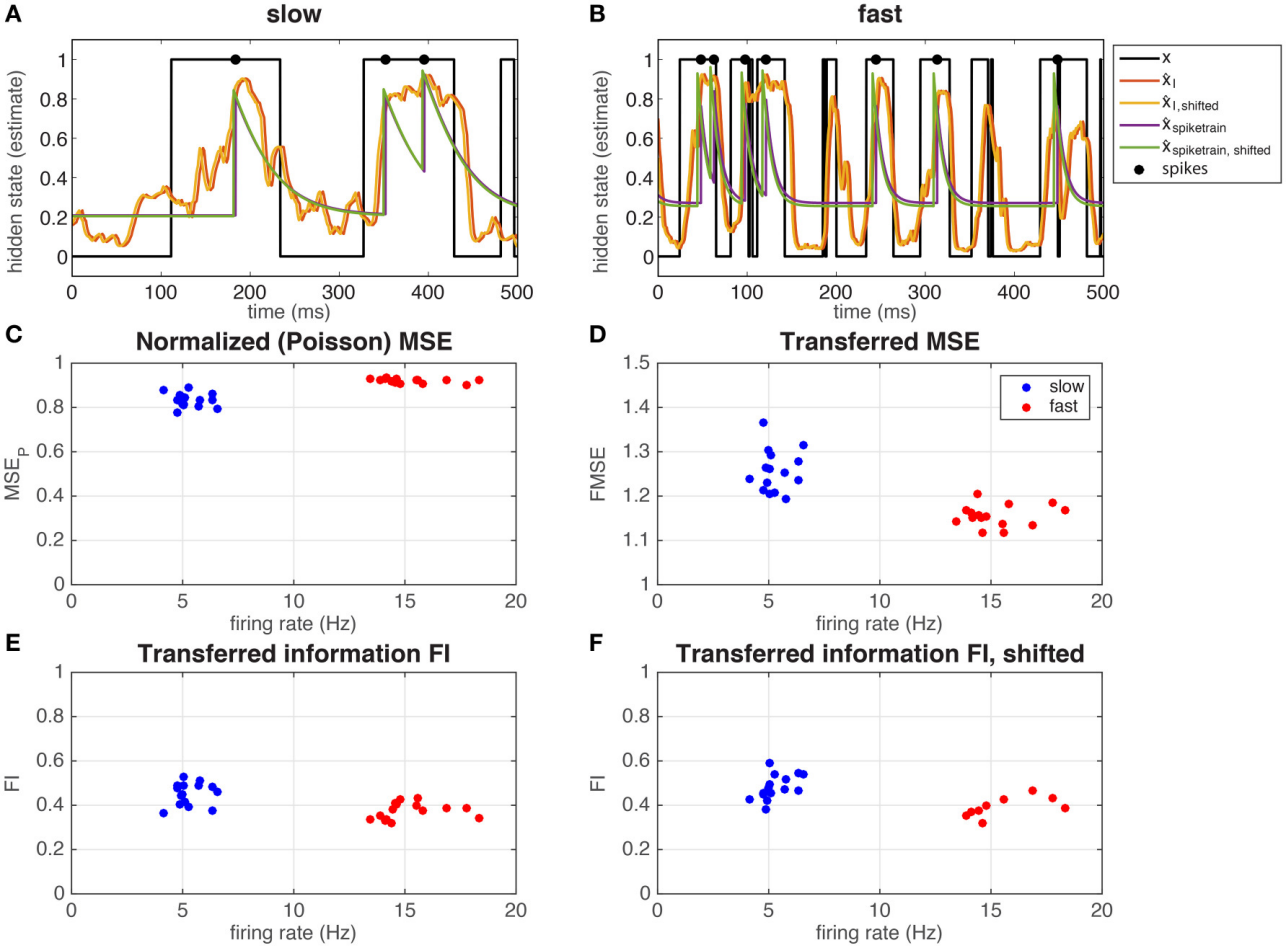
Representation of the hidden state by a single hippocampal pyramidal neuron (depicted with □ in Figure 5). The neuron responds to the input by spiking mostly when *x* = 1 in both the slow **(A)** and the fast **(B)** regime. The hidden state (black line) can be estimated from the input (red and yellow line) and from the output spike train (green and purple line), using a correction for the exponential kernel (yellow and green lines, section 2.1.2.6) or not (red and purple lines). In **(C)** the *MSE*_*P*_ (*MSE* normalized by a Poisson spike train, Equation 15) is shown as a function of the overall firing rate, and in **(D)** the *FMSE* (*MSE* of the spike train normalized by the *MSE* of the input, Equation 16). In **(E,F)**, we show the mutual information between the spike train and the hidden state normalized by the mutual information in the input, corrected for the exponential kernel **(F)** or not **(E)**.

**Figure 5:**
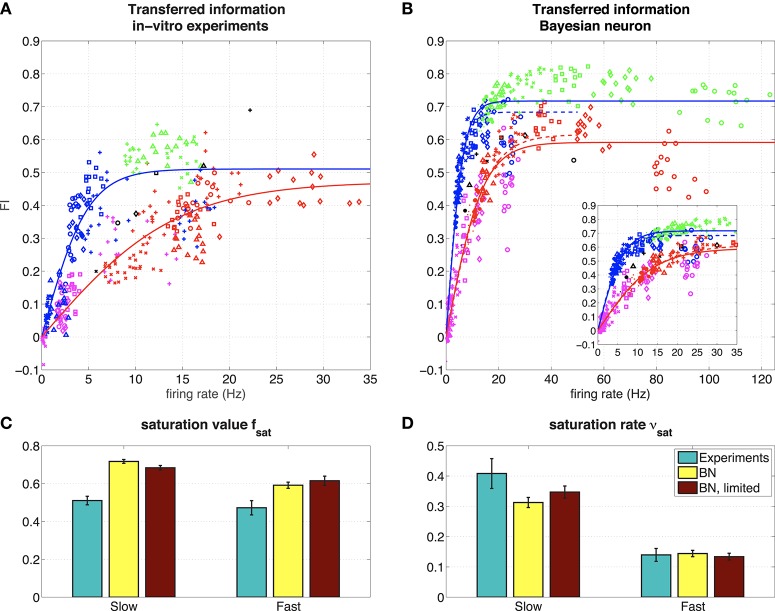
**(A)** Representation of the hidden state by 6 hippocampal pyramidal neurons (8 measurements). The figure shows the mutual information between the spike train and the hidden state normalized by the mutual information in the input, for 6 different hippocampal pyramidal neurons (depicted with symbols ◦, ◇, □, ×, +, △), in different regimes (blue: slow, red: fast, green: slow, high amplitude, pink: fast, low amplitude, black: probe). Solid lines: fit with saturating function (Equation 19). NB Note that even though theoretically *MI*_*I*_ ≥ 0 and *MI*_spike train_ ≥ 0, due to our approximation $\hat{M}I$ can take small negative values, and therefore so can *FI*. However, these effects are negligable (they only occur when both *MI*_*I*_ and *MI*_spike train_ are very small due to vanishing firing rates, which makes $FI\approx \frac{0}{0}$). **(B)** Representation of the hidden state by the Bayesian neuron (Section 2.1.3) for different values of the threshold/reset parameter η. The inset shows the same frequency range as in the experiments. Solid lines are fits using all data, dashed lines (“BN, limited”) are fits limited to values for η ≥ 2 (slow) or η ≥ 3 (fast). **(C)** Fitted parameters of saturating function (Equation 19) to data. Error bars denote 95 % confidence intervals of the fit.

In Figure 4 we show the hidden state and the different estimates of the hidden state (Equation 13), in the S (Figure 4A) and F (Figure 4B) regime, for a single hippocampal (CA1) pyramidal cell (depicted with □ in Figure 5). Note that in both regimes, spikes occur mostly in when *x* = 1, even if there is not a spike every time. In Figure 4C we calculated the *MSE* between the hidden state and the estimated hidden state, based on the spike times of the recorded neuron and normalized by a Poisson spike train of the same rate (*MSE*_*P*_, Equation 15). Note that the values in both the slow and fast regime are smaller than but not far from 1, meaning that the estimate is not much better than that of a Poisson process. The neuron performs slightly better in the slow regime: the difference in mean-squared error is small but significant (slow *MSE*_*P*_ = 0.83 ± 0.03, fast *MSE*_*P*_ = 0.92 ± 0.01, Student's *t*-test on difference *p* = 1.2·10^−7^). In Figure 4D it can be seen that the ratio between the *MSE* based on the spike train and the *MSE* based on the the input (Equation 16) is close to 1 (but significantly different; slow *FMSE* = 1.26 ± 0.05, Student's *t*-test on difference between 1: *p* = 5.8·10^−12^, fast *FMSE* = 1.16 ± 0.02, Student's *t*-test on difference between 1: *p* = 5.3·10^−13^). So even though the neuron does not perform much better than a Poisson process (Figure 4C), there is not much information loss between the input and the output spike train. The low mutual information between the spike train and the hidden state is a result of the low information content of the input. Indeed, in Figures 4E,F it is shown that the spike train transmits about 40–50% of the information in the input (Equation 3).

Even though the firing rate in the F regime is much higher than that of the S regime, the difference in output-information between the S and the F regime is very small [but significant: (Figure 4E) slow *FI* = 0.45 ± 0.05, fast *FI* = 0.37 ± 0.04, Student's *t*-test on difference *p* = 1.2·10^−4^, (Figure 4F) slow *FI*_shifted_ = 0.48 ± 0.06, fast *FI*_shifted_ = 0.39 ± 0.04, Student's *t*-test on difference *p* = 0.0012]. This means that the recorded neuron represent the hidden state states equally well in both regimes, but it is less *efficient* in the F state: it needs more spikes to transfer the same amount of information. As explained before (Section 3.1.1), more spikes are needed to represent a fast-switching hidden state. The result that the recorded neuron indeed increases its firing rate in the F regime relative to the S regime and the transferred information stays the same in both regimes suggests that the neuron “adapts” to the different regimes to keep the transferred information constant.

In Figure 5A we show the *FI* against the firing rate (same as in Figure 4E) for all recorded neurons. Different symbols denote different cells, whereas different colors denote the different regimes. The fraction of information about the hidden state in the input that is transmitted into the output spike train, depends on the amount of information in the input: in the very informative regime (SH, green), about 50–60% of the information in the input is transferred to the spike train, whereas in the low informative regime (FL, pink) only about 10% of the information is transmitted. In the intermediate S (blue), F (red), and P (black) regimes, the transmitted information is comparable and between these two extremes. The “adaptation” (the neuron transmits as much information in the S or F regime, but with different firing rates) seen in Figure 4 can be seen in 4 (◦, ◇, □, +) out of 6 neurons. The other two neurons (×, △) show a very low response in the slow state.

The firing rate of the neurons depends strongly on the amplitude of the input μ_*q*_: in the SH (green) and F (red) regime, that used the same value for μ_*q*_ (Table 1), the neurons show similar firing rates of around 15 Hz (except for a single neuron denoted with ◇). In the SH (pink) and S (blue) regime, which also used the same value for μ_*q*_ (Table 1), the neurons show low firing rates, with the firing rates of the FL regime, which has very little information about the hidden state in the input, having a lower artificial network firing rate. So the firing rates of the neurons increase with both the amplitude of the input and the amount of information.

#### 3.2.2. Comparison to an optimal response model

Finally, we compared the responses of the recorded neurons to a model of the optimal response for this input (Denève, 2008a; see Section 2.1.3). The parameters of this “Bayesian Neuron” (BN) are determined by the parameters of the input (i.e., *r*_on_, *r*_off_, and θ), except for parameter η, which determines the firing rate of the model neuron (changing η has a similar effect as changing the reset value and threshold in a leaky integrate-and-fire model).

In Figure 5B, we show how the BN performs in a simulation where we used the same input as we used in the experiments, for different values of η. Overall, the BN performs somewhat better than the recorded neurons, as can be expected from an optimal response model. However, as in the *in vitro* experiments, the BN increases its firing rate in the F state relative to the S state to keep fraction of transferred information relatively constant [compare for instance the F (red) and the S (blue) regime for η = 3.5, denoted with △].

In both the experiments and the simulations, the S and SH regimes seem to form a single curve, as do the F and FL regimes. In the Bayesian neuron this makes sense: the switching speed of the hidden state τ is a parameter of the model, the amplitude of the input μ_*q*_ is not. So the BN has the same parameters in the S and SH regimes, and the same is true for the F and FL regimes. The observation that these regimes also form a single curve in the experiments, suggest that the recorded neurons also adapt their response properties to the input statistics. The recorded and simulated neurons all transmit less information for a given firing frequency in the F and FL regimes than in the S and SH regimes, because in the F and FL regimes, more spikes are needed because more spikes are needed to represent a fast-switching hidden state.

For a quantitative comparison between the experiments and the BN, we fitted a saturating function to both slow states (green and blue, fits represented by blue lines) and both fast states (red and pink, fit represented by red lines) to the data from both the experiments and the model:

(19) $$\begin{array}{l} FI=2f_{\text{sat}}\left(\frac{1}{1+e^{-\nu_{\text{sat}}r}}-\frac{1}{2}\right), \end{array}$$

where *r* is the firing rate, *f*_sat_ is the saturation value and ν_sat_ the saturation rate (in s). Since the BN is an ideal observer model, we expect that the BN transmits more information than the experimentally measured neurons: we expect the saturation value *f*_sat_ to be higher, which is indeed what we find (Figure 5C). The closer the experimentally obtained *f*_sat_ is to the values from the model, the more “optimal” the information transfer of the hippocampal pyramidal cells.

Finally, for both the S and SH curve and the F and FL curve (Figure 5, right), there seems to be an optimal value for parameter η of the BN. This means that the BN appears to have an optimal firing rate: for too low firing rates (larger η) the neuron will miss some periods when *x* = 1, whereas for too high firing rates (smaller η) the neuron will also spike when *x* = 0, making the neuron less informative. This effect is stronger for the fast regimes than for the slow regimes. For all regimes investigated here, this optimal firing rate appears to be around 40 Hz (Figure 5B). In the experiments, we scaled the input current to set the firing rate response of the recorded neuron to the probe stimulus to about 12 Hz overall (about 20 Hz when *x* = 1), so this “optimal” firing rate of 40 Hz was never reached. Whether this 40 Hz is optimal for the recorded neurons too, remains to be investigated (see Grienberger et al., 2017 for natural firing regimes for hippocampal neurons).

## 4. Discussion

An important task of the brain is to infer information about the outside world. Except for sensory receptors, neurons in the brain do not have direct access to sources in the outside world, but have to infer the state of the world from input generated by other neurons. This input from other neurons is often unreliable and noisy (Knill and Richards, 1996; Körding and Wolpert, 2004). Therefore, neurons need enough input samples to keep a reliable estimate. The number of samples can be increased by either increasing the number of presynaptic neurons, or by integrating information over a longer period of time. Which one is feasible or appropriate depends on the characteristics of the local network (How many presynaptic neurons are available? With what frequency do they fire? How informative are they?) and on the characteristics of the outside world itself (How fast does a stimulus change?). Here, we modeled this by creating a current input for a single neuron that has to infer the presence or absence of a hidden state on the basis of noisy Poisson spike-trains of presynaptic neurons. Like in the general case, there is a trade-off between being fast, in which case many sources (pre-synaptic neurons) are needed, and being precise, in which case a longer integration time is needed, especially if there are not many presynaptic neurons. We propose to use the current stimulus designed here to measure in an *in vitro* setup how single neurons transfer information about a time varying stimulus.

We propose a new method to measure how much information a single neuron transfers from the (current) input it receives to the output spike train it generates. This method is based on generating current input as the response of an artificial population of presynaptic neurons responding to a stimulus randomly switching on and off, and measuring how well this hidden state can be constructed from the output spike train. This gives a lower bound on the mutual information between the spike train (Lochmann and Denève, 2008). This method has several advantages: (1) trials do not have to be repeated, since no estimate of the trial-to-trial variability is needed; (2) since no decoding model needs to be fitted, all recorded data can be used to measure the quantities of interest; (3) for comparison, the properties of an optimal response can be computed easily with the help of the Bayesian neuron (Denève, 2008a); (4) as the method is designed for an *in vitro* setup, stimuli are not limited to sensory stimuli, and neurons outside the sensory systems can be analyzed; (5) since we explicitly control how much information is present in the input, the information loss at the spike generating process itself can be measured; (6) experimental parameters, such as the “time constant of the world” and the number of available sources as discussed above can be systematically varied.

Like any method, the method presented here has several limitations and assumptions. We will discuss these explicitly. Firstly, three assumptions concern generating the input current for the experiments: (1) neurons respond to a randomly appearing and disappearing “preferred stimulus” that (2) they have no access to, and (3) synapses from informative presynaptic neurons are stronger than synapses from non-informative presynaptic neurons. The first two assumptions are comparable to the assumptions that are implicitly made when estimating tuning curves, for instance by fitting filter models such as a Linear–Non-linear Poisson model (Chichilnisky, 2001): in both cases it is assumed that a neuron responds only to the absence or presence (so no history or reverberation effects) of a preferred stimulus feature that it does not have direct access to. However, in the case of filter models, the presence of the preferred stimulus is graded: a preferred stimulus can be “more” or “less” present (i.e., the stimulus can be more or less similar to the preferred stimulus). Here, the stimulus is binary: it is either present or not. Which one is more realistic probably depends on the system in question. Whether the third assumption is realistic depends on the learning rule that was used by the system. Denève (2008b) showed that there exist indeed unsupervised, local, spike-based learning rules by which these synapse strengths could be learned. Secondly, the method requires two additional assumptions for the output spike trains: (1) an ergodic argument: it is assumed that an average over samples can be replaced by an average over time and (2) it is assumed that spike trains are by approximation Poissonian. The first assumption means that if in an experiment the system is not stationary during the time window for which the mutual information is calculated, the approximation fails. However, such an argument is necessary for almost any experimental measurement. Concerning the second assumption: the estimate of the mutual information is not strongly sensitive to how “Poissonian” the output spike train is, but strong deviations from Poissonian statistics will make the estimate fail. Finally, the fact that we used somatic patch-clamp stimulation, means that we ignored most of the computations that happen in dendritic trees, something that has proven to be substantial in hippocampal pyramidal cells (Spruston, 2008) and that could be essential for the integration of (correlated) inputs (Ujfalussy et al., 2015). This could be partly overcome by using bipolar electrodes and stimulate dendritically, for instance to evoke dendritic calcium spikes. However, the complex spatial distribution of dendritic inputs will be difficult to assess experimentally, although it could be investigated in a biophysical model. Another difference with the natural situation is that normally synaptic input creates conductance fluctuations, which have different (more complex) dynamics than the current injections we used in our model and experiments. For the moment we assume that this difference only creates second order differences.

We designed different input currents with the same amount of information about the hidden state, but with different switching speeds and firing rates (which are realistic for hippocampal neurons, see Grienberger et al., 2017), and injected these into the somata of pyramidal neurons in the CA1 region of mouse hippocampus. We found that the amount of information in the recorded spike trains depended strongly on the firing rate of the neuron: spike trains with more spikes were more informative about the hidden state than spike trains with fewer spikes. However, this effect saturated at around 15 Hz. The slope of the relationship between the firing rate and the mutual information depended on the switching speed of the hidden state: slowly changing inputs were easier to represent, hence contained more information for a given firing rate. However, the neurons responded to two inputs that contained comparable amounts of information about the hidden state, but had different characteristics (a “slow” input with a low amplitude and a “fast” input with a high amplitude) with different firing rates, but kept the amount of information in the recorded output spike trains constant, thereby “adapting^6^” to the characteristics of the stimulus. Strikingly, how much of the information about the hidden state in the input is transferred to the output spike train depended on how informative the input was in the first place: if the input was not very informative, not much information is transferred, whereas a much larger fraction of information about an informative input is transmitted to the output spike train, an effect that is also present in the optimal response of the Bayesian neuron, suggesting that biological neurons approximate an optimal inference process. So the spike-generating process of the recorded neurons has an amplifying effect on information transfer: it reduces the information about a low-informative input stronger than the information about a high-informative input (as explained in the Supplementary Material, the same holds for the relative signal-to-noise ratio: *FS* = *SNR*_output_/*SNR*_input_: the *FS* in response to an input with a low *SNR* is lower than the *FS* in response to an input with a high *SNR*).

The probability density functions of the membrane potential and the input current values show that that the input-current-to-membrane-potential transformation is strongly non-linear and could therefore not be described by for instance a simple leaky integrate-and-fire neuron. The strongly bimodal shape of the membrane potential distribution (as opposed to the input current distribution) can for instance be a result of a saturating (sigmoidal) input-output relation. From this non-linear processing and the amplifying effect on information transfer together we conclude that the neurons we recorded cannot have a simple linear input-output relation, but perform complex transformations on their input. In agreement with this conclusion, Ujfalussy et al. (2015) recently also suggested that the neural computation from presynaptic spikes to the postsynaptic membrane potential should be non-linear for optimal stimulus integration. How such non-linear input-output relationships shape the information processing properties of neurons and how they respond to stimuli with different characteristics (see also Stemmler and Koch, 1999; Brenner et al., 2000; Hong et al., 2008) remains an important topic that needs to be investigated further.

The mutual information between the position of an animal and the spike trains of rat hippocampal CA1 pyramidal cells has been quantified by Barbieri et al. (2004), who also used an estimate of the posterior probability to estimate the mutual information. They concluded that the hippocampal place cells contain a significant amount of information about the location of the animal. However, how much information was present in previous processing layers, and how much information is lost or maintained by these neurons, was not specified. Here, we quantified the information loss of the spike generating process, i.e., the mutual information between the cellular input and the output spike train. In barrel cortex, this information transfer has been quantified, and several studies have shown that spike generation can result in significant information loss (Panzeri et al., 2001; Petersen et al., 2002; Alenda et al., 2010), similar to what has been shown here. In hippocampus, *what* information is encoded in the spike trains has been described extensively since the discovery of place cells (O'Keefe and Dostrovsky, 1971). Moreover, *how* this information is encoded in the spike trains has been suggested to depend on the theta/gamma phase precession (Lisman, 2005). Finally, it has been shown that the nature of this information transfer (for instance the shape of place cell receptive fields) can change significantly, depending on for instance the age of the animal (Tanila et al., 1997). However, *how much* information is transferred by these cells, and how that depends on parameters such as the input characteristics, the state of the network (such as “up” or “down” states or the “high conductance state”; Destexhe et al., 2003) or the presence of neuromodulators such as dopamine or acetylcholine (ACh) remains to be quantified. Here, we provide a method to easily measure information transfer or information loss in hippocampus or any other system in an *in vitro* setup.

## Author contributions

FZ, SD, and BG designed the method. FZ, WW, and SdK designed the experiments. SdK performed the experiments. FZ wrote the manuscript.

### Conflict of interest statement

The authors declare that the research was conducted in the absence of any commercial or financial relationships that could be construed as a potential conflict of interest.

## Footnotes

^1^Note that increasing the number of presynaptic neurons or the firing rates of the presynaptic neurons has the same effect: increasing the stimulus amplitude relative to the background noise. This relative stimulus amplitude is related to, but not the same as, the signal-to-noise ratio (see Supplementary Material).

^2^For large enough *N*, θ ≈ 0. Since $q_{\text{on}}^{i}$ and $q_{\text{off}}^{i}$ are drawn from the same normal distribution with mean μ_*q*_ and standard deviation $\sigma_{q}=\sqrt{\frac{1}{8}}\mu_{q}$, the difference distribution has mean 0 and standard deviation $\sqrt{2}\sigma_{q}=\sqrt{\frac{1}{2}}\mu_{q}$.

^3^Due to the discretization of numerical approaches, “true” δ-spikes cannot be implemented in a computer. Rather, a δ-spike is implemented as a square kernel with width *dt* and height $\frac{1}{dt}$.

^4^Note that this gives us another estimate of the mutual information, based on the relation between the signal-to-noise ratio and the mutual information $MI=\int_{0}^{\infty }\log_{2}\left(1+SNR\left(f\right)\right)df$ (Shannon, 1984; Cover and Thomas, 1991; Guo et al., 2005; Schultz, 2007) and the noise signal is defined as noise $=x-\hat{x}$. However, this will give us essentially the same results, since it is based on the same estimate $\hat{p}\left(x=1\right)$ and $\hat{L}$.

^5^If we would use delta-spikes to simulate postsynaptic current shapes these two options are completely equivalent (as long as *N* is large enough, about 100 or more neurons are needed or otherwise the realization of the firing rates and therefore the weights *w* results in large variations between realizations). For all other PSC-shapes, there could be a small effect if overlapping spikes from different neurons are added different from overlapping spikes from the same neuron, but we will not consider this technical issue here, since the effects are small.

^6^The word “adapting” is between quotation marks, since it is possible that this effect is caused by non-linear but instantaneous processes in the neuron and not by an active adaptive process, compare for instance to Hong et al. (2008).

## Abbreviations

BN: Bayesian Neuron, see Denève (2008a)
MSE: Mean-Squared Error (Equation 14)
FMSE: Fraction of output MSE relative to input MSE (Equation 16)
MSE_*P*_: Fraction of output MSE relative to MSE in Poisson spike train (Equation 15)
F: Fraction of information relative to entropy of the hidden state (Equation 2)
FI: Fraction of information about the hidden state in output relative to input (Equation 3)
FS: *SNR*_output_/*SNR*_input_.
