# Supplementary material for: Estimating the Information Extracted by a Single Spiking Neuron from a Continuous Input Time Series
Source: Front Comput Neurosci. 2017 Jun 15;11:49. doi: 10.3389/fncom.2017.00049 (PMC5471316; doi:10.3389/fncom.2017.00049)
Supplement: Supplementary file 1 [file DataSheet1.pdf]

Supplementary Material: Estimating information loss between input and spike train

Fleur Zeldenrust [1], Sicco de Knecht [2], Sophie Denève [3], Boris Gutkin [3] and Wytse J. Wadman [2]

*Correspondence:
Fleur Zeldenrust
f.zeldenrust@neurophysiology.nl

1 SUPPLEMENTARY TABLES AND FIGURES

1.1 Relative Stimulus Amplitude (RSA) and autocorrelation time-constant

Even though the time constant of the hidden state (τ) and the firing rates of the presynaptic neurons (μ_q) have a similar effect on the mutual information between the hidden state and the input, their effects on the shape of the input are quite different: the effect of increasing μ_q is that the amplitude of the stimulus increases and that the difference in amplitude between when v$x = 1$) and the when $x = 0$ relative to the standard deviation increases. We define this quantity as the Relative Stimulus Amplitude (RSA) (see equation (S1)). Alternatively, increasing τ does not increase the RSA, but changes the autocorrelation-time τ_{auto} of the input signal. So, with τ and μ_q we can vary the stimulus amplitude (RSA) and autocorrelation-time independently, while keeping the mutual information between the stimulus and the hidden state constant. Here we will use the following two quantities to charachterize the stimulus:

- We define the **Relative Stimulus Amplitude (RSA)** as the difference between the difference in mean input in the 'up state' and 'down state' divided by the average standard deviation of the input [1]:

$$\text{RSA} = \frac{\langle I \rangle_{\text{time in up state}} - \langle I \rangle_{\text{time in down state}}}{(\sigma(I)_{\text{time in up state}} + \sigma(I)_{\text{time in down state}})/2}. \tag{S1}$$

- We measure the **autocorrelation time-constant τ_{auto}** by measuring where the autocorrelogram of the input has decayed to 36,8 % (e^{-1}) of its maximal value.

In figure S1 we show both the RSA and the measured τ_{auto} of the generated input as a function of τ and μ_q. As expected, τ_{auto} has a minimum value of 5 ms, due to the exponential kernel mimicking the PSC-shape. Note that for small τ, the RSA is not independent from τ: the input is always slightly delayed due to the exponential kernel, so for small τ the RSA cannot reliably be measured. Similarly, the measured τ_{auto} will always be a mixture between the autocorrelation-time of the hidden state (τ) and the decay-time of the exponential kernel we used to mimick PSC shapes (5 ms). The weight of each of these in the mix, depends on both μ_q and τ. Therefore, τ_{auto} will never take values smaller than 5 ms, and will not be completely independent of μ_q.

[1] Note that the RSA is not the signal-to-noise ratio (SNR), which is defined as the *power* of the signal divided by the *power* of the noise. However, the RSA is related to the SNR, as naturally for our stimulus the RSA does increase with the SNR.

1.2 Relation with Signal-to-Noise Ratio

For a discrete time channel with additive Gaussian noise, the following relation between the Signal-to-Noise Ratio (SNR) and the mutual information (MI) can be expressed **?**:

$$MI = \frac{1}{2}\log_2(1 + SNR) \tag{S2}$$

This gives the following relation between the ratio of MI in the input and output and the SNR in the input and output:

$$\frac{MI_{\text{output}}}{MI_{\text{input}}} = FI = \frac{\log_2(1 + SNR_{\text{output}})}{\log_2(1 + SNR_{\text{input}})} \tag{S3}$$

Assuming that the mutual information between the input and the hidden state is about 0.2 bits (see figure 2 of the main text, given that the entropy of the hidden state is 0.91 bits), the relation between FI and the SNR ratio $FS = \frac{SNR_{\text{output}}}{SNR_{\text{input}}}$ is shown in figure **??**. Note that for such a discrete Gaussian channel, the relation between FS and FI is almost linear, and that FS and FI are almost identical. Based on this, we claim that our conclusions for FI hold for FS too.

1.3 Figures

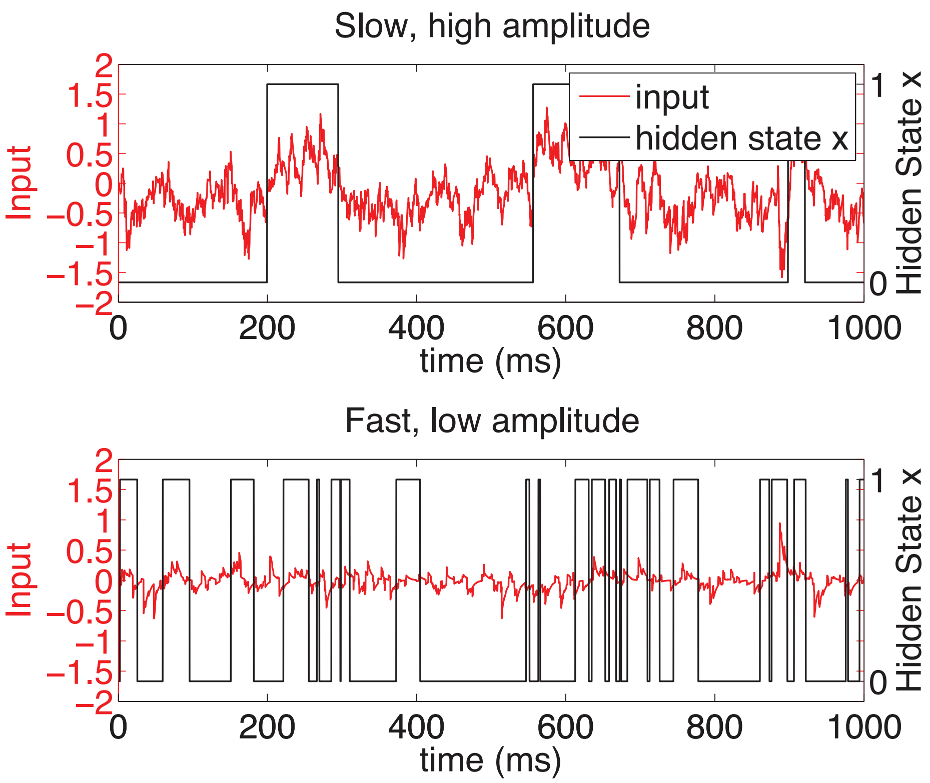

Figure S1. Example of a realization of the hidden state (black) and the input from the artificial presynaptic neurons (red) in the 'slow switching - high amplitude' (SH) regime (top) and the 'fast switching - low amplitude' (FL) regime (bottom).

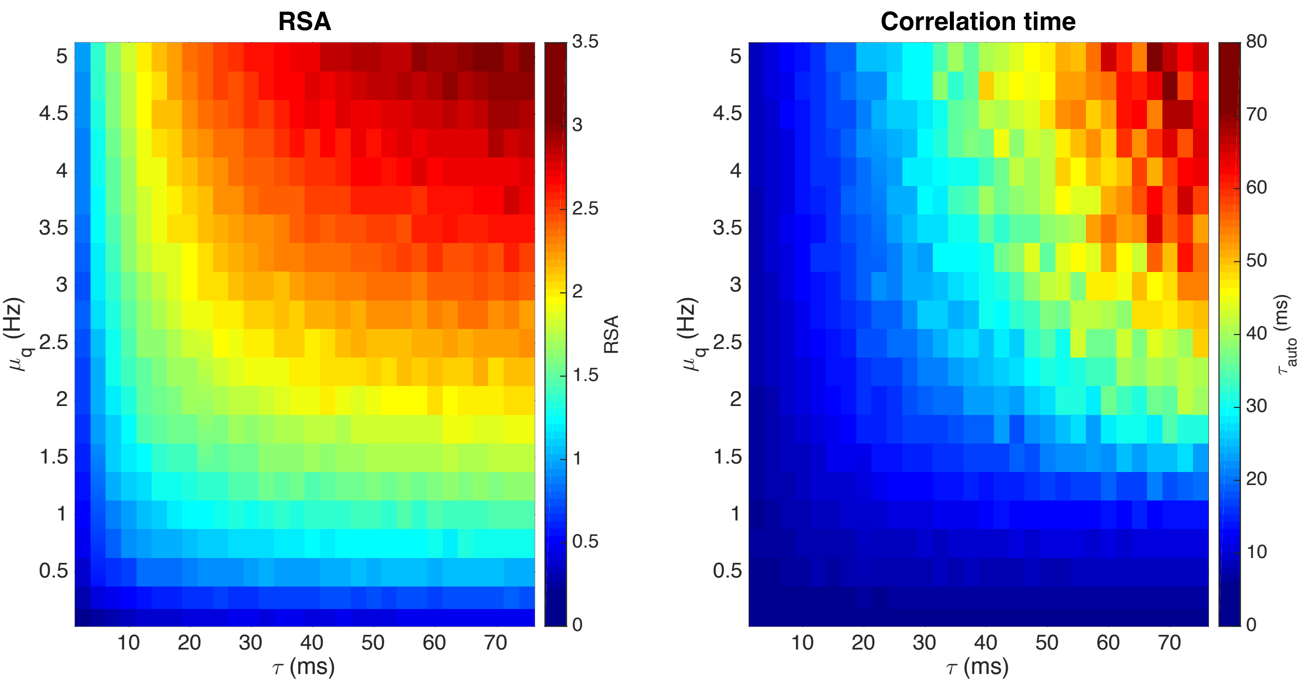

Figure S2. The Relative Stimulus Amplitude (RSA, equation (S1)) and autocorrelation time-constant (τ_{auto}) of the input generated by the network of $N = 1000$ artificial neurons as a function of the firing rate of the neurons in the network (μ_q) and the switching speed (time constant τ) of the hidden state.

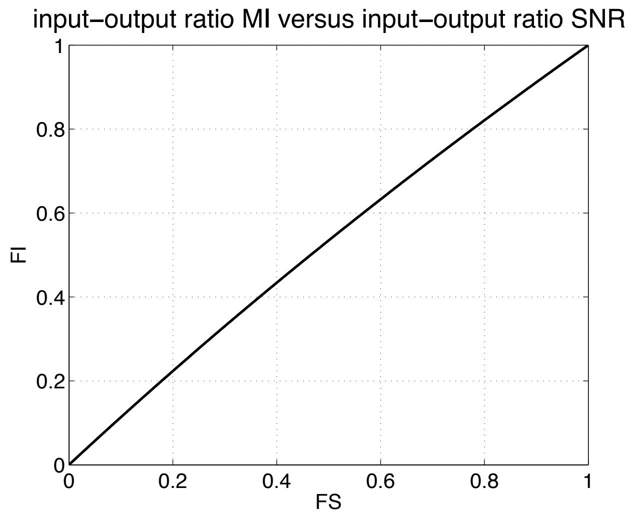

Figure S3. The SNR ratio FS as a function of the information ratio FI, assuming a discrete time channel with additive Gaussian noise and a mutual information between the input and the hidden state $MI_{\text{input}} = 0.2$ bits.
